# Proximal Pathway Enrichment Analysis for Targeting Comorbid Diseases via Network Endopharmacology

**DOI:** 10.3390/ph11030061

**Published:** 2018-06-22

**Authors:** Joaquim Aguirre-Plans, Janet Piñero, Jörg Menche, Ferran Sanz, Laura I. Furlong, Harald H. H. W. Schmidt, Baldo Oliva, Emre Guney

**Affiliations:** 1 Research Programme on Biomedical Informatics, the Hospital del Mar Medical Research Institute and Pompeu Fabra University, Dr. Aiguader 88, 08003 Barcelona, Spain; joaquim.aguirre@upf.edu (J.A.-P.); janet.pinero@upf.edu (J.P.); ferran.sanz@upf.edu (F.S.); laura.furlong@upf.edu (L.I.F.); baldo.oliva@upf.edu (B.O.); 2 CeMM Research Center for Molecular Medicine of the Austrian Academy of Sciences, Lazarettgasse 14, AKH BT 25.3, A-1090 Vienna, Austria; JMenche@cemm.oeaw.ac.at; 3 Department of Pharmacology and Personalised Medicine, CARIM, FHML, Maastricht University, Universiteitssingel 50, 6229 ER Maastricht, The Netherlands; h.schmidt@maastrichtuniversity.nl

**Keywords:** drug repurposing, proximal pathway enrichment analysis, network endopharmacology, systems medicine, comorbidity, autoimmune disorders, Alzheimer’s disease, type 2 diabetes

## Abstract

The past decades have witnessed a paradigm shift from the traditional drug discovery shaped around the idea of “one target, one disease” to polypharmacology (multiple targets, one disease). Given the lack of clear-cut boundaries across disease (endo)phenotypes and genetic heterogeneity across patients, a natural extension to the current polypharmacology paradigm is to target common biological pathways involved in diseases via endopharmacology (multiple targets, multiple diseases). In this study, we present proximal pathway enrichment analysis (PxEA) for pinpointing drugs that target common disease pathways towards network endopharmacology. PxEA uses the topology information of the network of interactions between disease genes, pathway genes, drug targets and other proteins to rank drugs by their interactome-based proximity to pathways shared across multiple diseases, providing unprecedented drug repurposing opportunities. Using PxEA, we show that many drugs indicated for autoimmune disorders are not necessarily specific to the condition of interest, but rather target the common biological pathways across these diseases. Finally, we provide high scoring drug repurposing candidates that can target common mechanisms involved in type 2 diabetes and Alzheimer’s disease, two conditions that have recently gained attention due to the increased comorbidity among patients.

## 1. Introduction

Following Paul Ehrlich’s more-than-a-century-old proposition on magic bullets (one drug, one target, one disease), the drug discovery pipeline traditionally pursues a handful of leads identified in vitro based on their potential to bind to target(s) known to modulate the disease [1]. The success of the selected lead in the consequent clinical validation process relies on the prediction of a drug’s effect in vivo. Although it is often more desirable to tinker the cellular network by targeting multiple proteins [2], this is hard to achieve in practice due to the interactions of the compound and its targets with other proteins and metabolites. As a result, the characterization of drug effect has been a daunting task, yielding high pre-clinical attrition rates for novel compounds [3,4].

The high attrition rates can be attributed to the immense response heterogeneity across patients, likely stemming from a polygenic nature of most complex diseases. Consequently, researchers have turned their attention to polypharmacology, where novel therapies aim to alter multiple targets involved in the pathway cross-talk pertinent to the disease pathology, rather than single proteins [5,6]. This has given rise to network-based approaches that predict the effects of individual drugs [7] as well as drug combinations [8], allowing for the repositioning of compounds for novel indications.

Over the past years, reusing existing drugs for conditions different from their intended indications has emerged as a cost effective alternative to traditional drug discovery. Various drug repurposing methods aim to mimic the most likely therapeutic and safety outcomes of candidate compounds based on similarities between compounds and diseases characterized by high-throughput omics data [9,10,11]. Most studies so far, however, have focused on repurposing drugs for a single condition of interest, failing to recognize the cellular, genetic and ontological complexity inherent to human diseases [12,13]. In reality, pathway cross-talk plays an important role in modulating the pathophysiology of diseases [14] and most comorbid diseases are interconnected to each other in the interactome through proteins belonging to similar pathways [15,16,17,18,19]. The pathway cross-talk is especially relevant for autoimmune disorders, which have been shown to share several biological functions involved in immune and inflammatory responses [20,21]. Autoimmune disorders affect around 15% of the population in the USA [22] and co-occur in the same patient more often than expected (i.e., comorbid) [23]. Recent evidence suggests that endophenotypes—shared intermediate pathophenotypes—[24], such as inflammasome, thrombosome, and fibrosome play essential roles in the progression of not only autoimmune disorders but also many other diseases [25].

Here, we propose a novel drug repurposing approach, **P**ro**x**imal pathway **E**nrichment **A**nalysis (PxEA), to specifically target intertangled biological pathways involved in the common pathology of complex diseases. We first identify pathways proximal to disease genes across various autoimmune disorders. Then we use PxEA to investigate whether the drugs promiscuously used in these disorders target specifically the pathways associated with one disease or the pathways shared across the diseases. We find several examples of anti-inflammatory drugs where the pathways proximal to the drug targets in the interactome correspond to the pathways shared between two autoimmune disorders. The observed lack of specificity among these drugs points to the existence of immune system related endophenotypes, motivating us to explore shared disease mechanisms for repurposing drugs. We demonstrate that PxEA is a powerful computational strategy for targeting multiple pathologies involving common biological pathways, such as type 2 diabetes (T2D) and Alzheimer’s disease (AD). Based on these findings, we argue that PxEA paves the way for simultaneously targeting endophenotypes that manifest across various diseases, a concept which we refer to as *endopharmacology*.

## 2. Results

### 2.1. Pathway Proximity Captures the Similarities between Autoimmune Disorders

Conventionally, functional enrichment analysis relies on the significance of the overlap between a set of genes belonging to a condition of interest and a list of genes involved in known biological processes (pathways). Using known pathway genes, one can identify pathways associated with the disease via a statistical test (e.g., Fisher’s exact test for the overlap between genes or z-score comparing the observed number of common genes to the number of genes one would have in common if genes were randomly sampled from the data set). We start with the observation that such an approach (hereafter referred as to *conventional* approach) often misses key biological processes involved in the disease due to the limited overlap between the disease and pathway genes. To show that this is the case, we focus on nine autoimmune disorders for which we obtain genes associated with the disease in the literature and we calculate *p*-values based on the overlap between these genes and the pathway genes for each of the 674 pathways in the Reactome database (Fisher’s exact test, one-sided p≤0.05). Intriguingly, Table 1 demonstrates that this conventional approach yields less than ten pathways that are significantly enriched in five out of nine diseases, potentially underestimating the molecular underpinning of these diseases.

Alternatively, the shortest distance between genes in the interactome can be used to find pathways closer than random expectation to a given set of genes [7,26], augmenting substantially the number of pathways relevant to the disease pathology. Using network-based proximity [7], we define the *pathway span* of a disease as the set of pathways significantly proximal to the disease (z≤−2, see Methods). We show that the number of pathways involved in diseases increases substantially when proximity is used (Table 1).

**Figure 1:**
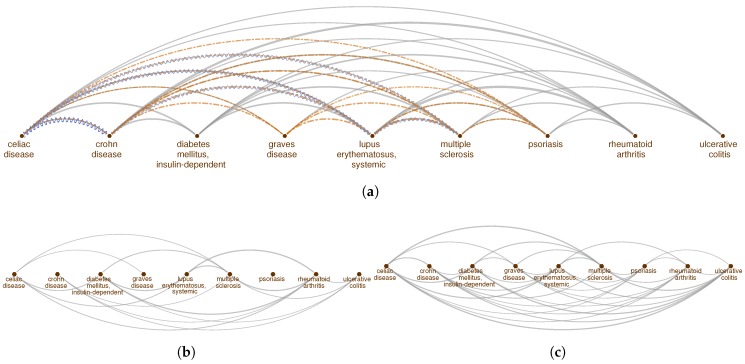
Genetic, phenotypic and functional overlap across autoimmune disorders. Disease relationships (links) based on (**a**) shared genes (gray solid lines), shared symptoms (orange dashed lines) and comorbidity (blue sinusoidal lines); (**b**) shared pathways (gray solid lines) using common disease and pathway genes, (**c**) shared pathways (gray solid lines) using the proximity of the pathway genes to the diseases genes in the interactome.

To show the biological relevance of the identified pathways using interactome-based proximity, we check how well these pathways can highlight genetic and phenotypic relationships between nine autoimmune disorders. First, to serve as a background model, we build a disease network for the autoimmune disorders (diseasome) using the genes and symptoms shared between these diseases as well as the comorbidity information extracted from medical insurance claim records (see Methods). The autoimmune diseasome (Figure 1a) is extremely connected, covering 33 out of 36 potential links between nine diseases (with average degree <k> = 7.3 and clustering coefficient CC=0.93). The three missing links are those between ulcerative colitis and rheumatoid arthritis, ulcerative colitis and Graves’ disease, and Graves’ disease and type 1 diabetes. On the other hand, several diseases such as celiac disease, Crohn’s disease, systemic lupus erythematosus, and multiple sclerosis are connected to each other with multiple evidence types in the autoimmune diseasome based on genetic (shared genes) and phenotypic (shared symptoms and comorbidity) similarities, emphasizing the shared pathological components underlying these diseases.

We compare the autoimmune diseasome generated using shared genes, common symptoms and comorbidity, to the disease network in which the disease-disease connections are identified using the pathways they share. We identify the pathways enriched in the diseases using both the conventional and proximity approaches mentioned above and check whether the number of common pathways between two diseases is significant (two-tailed Fisher’s exact test, p<0.05). The disease network based on pathways shared across diseases using the overlap between the pathway and disease genes is markedly sparser than the original diseasome, containing 17 links (Figure 1b). None of the diseases share pathways with psoriasis and among the connections supported by multiple evidence in the original diseasome, the links between Crohn’s disease and celiac disease as well as Crohn’s disease and systemic lupus erythematosus are missing. On the contrary, the disease network based on shared pathways using proximity of the pathway genes to the disease genes consists of 34 links, where the only unconnected disease pairs are Crohn’s disease and Graves’ disease and type 1 diabetes and psoriasis, suggesting that it captures the connectedness of the original diseasome better than the conventional approach.

We next turn our attention to the shared pathways across diseases identified by both conventional and proximity based approaches and observe that most common pathways involve biological processes relevant to the immune system endophenotypes. In particular, we see that inflammasome-related pathways, such as signaling of cytokines (interferon gamma, interleukins like IL6, IL7) and lymphocytes (ZAP70, PD1, TCR, among others) are overrepresented. While conventional enrichment finds that most of these pathways are shared among only 4–5 diseases, proximity based enrichment points to the commonality of these pathways among almost all the diseases. Furthermore, the proximity based enrichment uncovers the involvement of additional interleukin (IL2, IL3, IL5) and lymphocyte (BCR) molecules ubiquitously in autoimmune disorders. These findings suggest that proximity-based pathway enrichment identifies biological processes relevant to the diseases, highlighting the common etiology across autoimmune disorders.

### 2.2. Diseases Targeted by the Same Drugs Exhibit Functional Similarities

**Figure 2:**
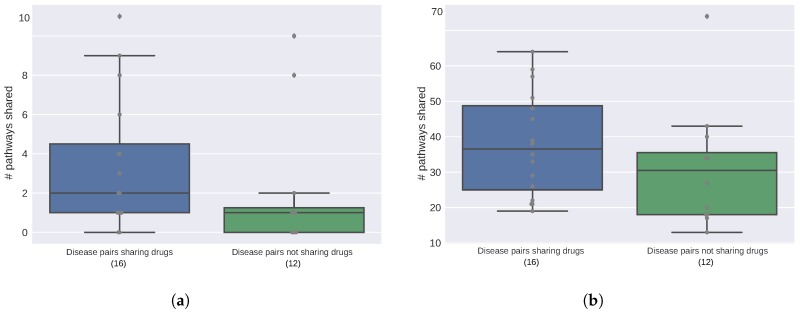
Number of shared pathways across disease pairs that are targeted by the same drug compared to the rest of the pairs. The pathway enrichment is calculated using (**a**) gene overlap and (**b**) proximity of genes in the interactome. The number of disease pairs in each group is given in the parenthesis below the group label in the *x*-axis.

Having observed that pathway proximity to diseases in the interactome captures the underlying biological mechanisms across diseases, we seek to investigate the potential implications of the connections between diseases for drug discovery. We hypothesize that a drug indicated for several autoimmune disorders would exert its effect by targeting the shared biological pathways across these diseases. To test this, we use 25 drugs that are indicated for two or more of the autoimmune disorders in Hetionet [27] and split disease pairs into two groups: (i) diseases for which a common drug exists and (ii) diseases for which no drugs are shared. We then count the number of pathways in common between two diseases for each pair in the two groups using pathway enrichment based on both the gene overlap and proximity in the interactome. We find that the diseases targeted by the same drugs tend to involve an elevated number of common pathways compared to the disease pairs that do not have any drug in common (Figure 2). The average number of pathways shared among diseases that are targeted by the same drug is 3.4 and 38 using overlap and proximity based enrichment, respectively, whereas, the remaining disease pairs share 2 and 31 pathways on average using the two enrichment approaches. We note that due to the relatively small sample size and potentially incomplete drug indication information, we interpret the elevated number of pathways as a trend rather than a general rule across all diseases (p=0.043 and p=0.066, assessed by one-tailed Mann-Whitney U test, for the overlap and proximity based approaches, respectively). Nevertheless, taken together with the high overall pathway level commonalities observed in the autoimmune disorders mentioned in the previous section, this result suggests that the drugs used for multiple indications are likely to target common pathways involved in these diseases.

### 2.3. Proximal Pathway Enrichment Analysis Reveals Drugs Targeting the Autoimmune Endophenotypes

**Figure 3:**
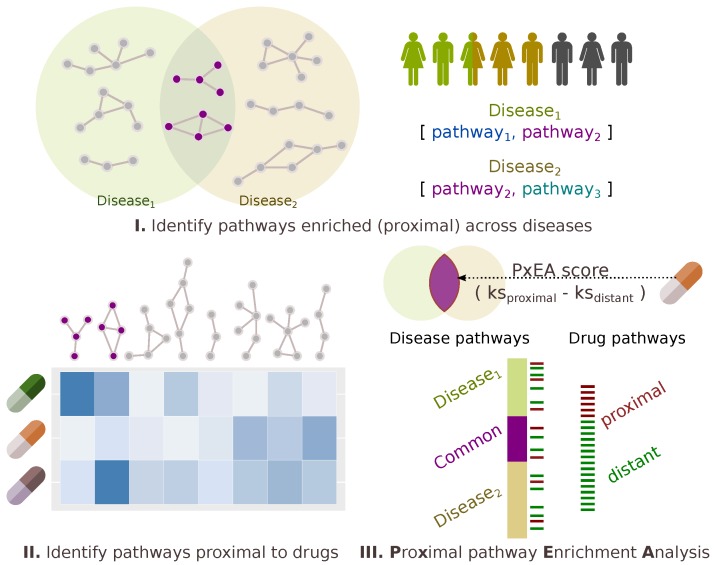
Schematic overview of proximal pathway enrichment analysis (PxEA). PxEA scores a drug with respect to its potential to target the pathways shared between two diseases. For a given drug and two diseases of interest, PxEA first identifies the common pathways between the two diseases and then uses the proximity-based ranking of the pathways (i.e., average distance in the interactome to the nearest pathway gene, normalized with respect to a background distribution of expected scores) to assign a score to the drug and the disease pair.

The results indicating that the drugs used for multiple autoimmune disorders potentially target common pathways raise the following question: “Can pathway level commonalities between diseases be leveraged to quantify the impact of a given drug on these diseases?” To this end, we propose PxEA, a novel method for **P**ro**x**imal pathway **E**nrichment **A**nalysis that scores the likelihood of a set of pathways (e.g., targeted by a drug) to be represented among another set of pathways (e.g., disease pathways) based on the proximity of the pathway genes in the interactome. As opposed to the Gene Set Enrichment Analysis (GSEA) [28] which uses gene sets and the ranking of genes based on differential expression, PxEA uses pathway sets and the ranking of pathways based on proximity in the interactome. PxEA scores a drug based on whether or not the pathways targeted by the drug are proximal to a pathway set of interest, such as pathways shared across different diseases. For a given drug and a pair of diseases, we first identify the pathways in the pathway span of both of the diseases, then we rank the pathways with respect to the proximity of the drug targets to the pathway genes and finally we calculate a running sum statistics corresponding to the enrichment score between the drug and the disease pair (Figure 3, see Methods for details).

**Figure 4:**
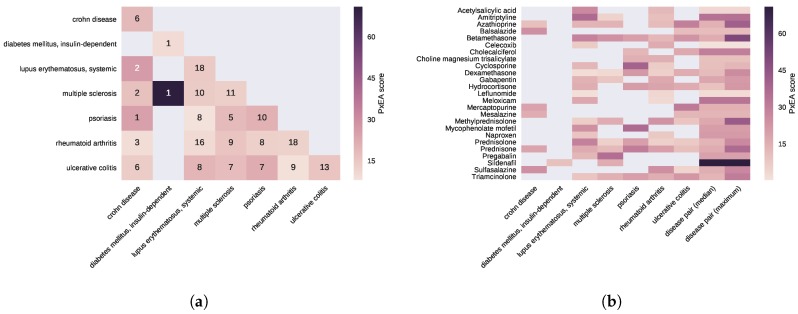
PxEA scores of drugs used in autoimmune disorders. (**a**) Disease-disease heatmap, in which for each disease pair, the common pathways proximal to the two diseases are used to run PxEA. Note that the diagonal contains the PxEA scores obtained when the proximal pathways for only that disease are used. The hue of the color scales with the PxEA score. (**b**) Drug-disease heatmap, in which the PxEA is run using the pathways proximal to the pathways of the disease in the column for the drugs in the rows (25 drugs that are used at least in two diseases). The last two columns show the median and maximum values of the PxEA scores obtained for the drug among all disease pairs the drug is indicated for.

We employ PxEA to score 25 drugs indicated for at least two of the seven autoimmune disorders (there were no common drugs for celiac and Graves’ diseases). For each disease, we first run PxEA using the pathways proximal to the disease and the proximity of the drugs used for that disease to these pathways. We then run PxEA for each disease pair, using the pathways proximal to both of the diseases in the pair and the drugs commonly used for the two diseases. We notice that several drugs indicated for multiple conditions score higher using common pathways between two diseases than using the pathways of the disease they are indicated for (Figure 4). This is not surprising considering that many of the drugs used for autoimmune disorders target common immune and inflammatory processes. For instance, sildenafil, a drug used for the treatment of erectile dysfunction and to relieve the symptoms of pulmonary arterial hypertension, is reported by Hetionet to show palliative effect on type 1 diabetes and multiple sclerosis. Actually, sildenafil is not specific to any of these two conditions and targets a number of the 57 pathways in common between type 1 diabetes and multiple sclerosis including but not limited to pathways mentioned in Table 2, such as “IL-3, 5 and GM CSF signaling” (z=−1.6), “regulation of signaling by CBL” (z=−1.1), “regulation of KIT signaling” (z=−1.0), “IL receptor SHC signaling” (z=−1.0), and “growth hormone receptor signaling” (z=−1.0).

Similarly, prednisone, a synthetic anti-inflammatory glucocorticoid agent that is indicated for six of the autoimmune disorders, is assigned a higher PxEA score using the pathways shared by Crohn’s disease and systemic lupus erythematosus compared to using the pathways involved only in Crohn’s disease, systemic lupus erythematosus, multiple sclerosis, psoriasis, rheumatoid arthritis, or ulcerative colitis. Thus, prednisone does not specifically target any of the six autoimmune disorders but rather acts on the endophenotypes that manifest across these diseases. We observe a similar trend in meloxicam, an anti-inflammatory drug that shows analgesic and antipyretic effects by inhibiting prostaglandin synthesis. Consistent with its known mechanism of action, meloxicam is proximal to “cholesterol biosynthesis” (z=−3.5), “fatty acid, triacylglycerol, and ketone body metabolism” (z=−2.0), and “prostanoid ligand receptors” (z=−1.7) pathways in the interactome. While meloxicam is originally indicated for rheumatoid arthritis and systemic lupus erythematosus, the higher PxEA score when common arthritis and lupus pathways are used suggests that it targets common inflammatory processes in these two diseases.

### 2.4. Targeting the Common Pathology of Type 2 Diabetes and Alzheimer’s Disease

T2D and AD, two diseases highly prevalent to an ageing society, are known to exhibit increased comorbidity [29,30]. Recently, repurposing anti-diabetic agents to prevent insulin resistance in AD has gained substantial attention due to the therapeutic potential it offers [31]. Indeed, the pathway spans of T2D and AD cover 170 and 82 pathways, respectively, 35 of which are shared between two diseases, linking significantly the two diseases at the pathway level (Fisher’s exact test, two-sided $p=2.2\times 10^{-4}$).

**Figure 5:**
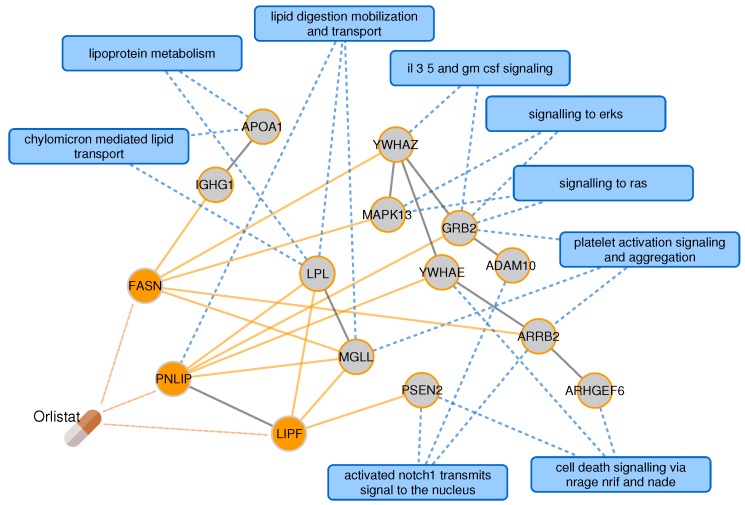
Orlistat from PxEA perspective. The subnetwork shows how the targets of Orlistat are connected to the nearest pathway protein for the pathways shared between T2D and AD. For clarity, only the pathways that are proximal to the drug are shown. Blue rectangles represent pathways, circles represent drug targets (orange) or proteins on the shortest path to the nearest pathway gene (gray). Blue dashed lines denote pathway membership, solid lines are protein interactions. The interactions between the drug and its targets are shown in dashed orange lines and the interactions between the drug targets and their neighbors are highlighted with solid orange lines.

We use PxEA to score 1466 drugs from DrugBank using the 35 pathways involved in the common pathology of T2D and AD. When we look at the drugs ranked on the top of the list (Table 3), we spot orlistat, a drug indicated for obesity and T2D in Hetionet. Interestingly, existing studies also suggest a role for this drug in the treatment of AD [32]. Orlistat targets extracellular communication (Ras-Raf-MEK-ERK, NOTCH, and GM-CSF/IL-3/IL-5 signaling) and lipid metabolism pathways (Figure 5). Several of the proteins in the pathways pertinent to the common T2D-AD pathology, such as APOA1, PSEN2, PNLIP, LPL, and IGHG1 are either Orlistat’s targets themselves or are in the close vicinity of the targets. The next top scoring drugs are chenodeoxycholic and obeticholic acid, biliar acids that are in clinical trials for T2D (NCT01666223) and are argued to modulate cognitive changes in AD [33].

It is noteworthy that the top scoring drugs belong to a diverse set of Anatomical Therapeutic Chemical (ATC) classes, covering alimentary tract and metabolism drugs (A05, A06, A08, A12), blood substitutes (B05), dermatologicals (D11) as well as cardiovascular (C01, C07), genito-urinary (G02), nervous (N07), and respiratory (R03) system drugs. The diversity of the ATC classes of top scoring drugs indicates that PxEA is not biased towards any particular ATC class. We also calculate the significance of the PxEA scores by permuting the ranking of the pathways. We find that the adjusted *p*-values (corrected for multiple hypothesis testing using Benjamini–Hochberg procedure) for the top candidates are all below $1\times 10^{-4}$, the minimum possible value (due to the 10,000 permutations used in the calculation).

## 3. Discussion

The past decades have witnessed a substantial increase in human life expectancy owing to major breakthroughs in translational medicine. Yet, the increase on average age and changes in life style, have given rise to a spectra of problems challenging human health like cancer, neurodegenerative disorders and diabetes. These diseases do not only limit the life expectancy but also induce a high burden on public healthcare costs. In the US alone, more than 20 and 5 million people have been affected by T2D and AD, respectively, ranking these diseases among the most prevalent health problems [29].

Mainly characterized by hyperglycemia due to resistance to insulin, the disease mechanism of T2D involves a combination of multiple genetic and dietary factors. On the other hand, AD is relatively less understood and several hypotheses have been proposed for its cause: reduced synthesis of neurotransmitter acetylcholine, accumulation of amyloid beta plaques and/or tau protein abnormalities, giving rise to neurofibrillary tangles. Accordingly, most available treatments in AD are palliative (treating symptoms rather than the cause). Given the comorbidity between T2D and AD [29,30] several studies have recently suggested repurposing diabetes drugs for AD [31]. However, to our knowledge, currently there is no systematic method that can pinpoint drugs that could be useful to target common disease pathology such as the one between T2D and AD.

In this study, we first show that diseases that share drugs also tend to share biological pathways and hypothesize that these pathways can be targeted to exploit novel drug repurposing opportunities. We introduce PxEA, a method based on (i) pathways that are proximal to diseases and (ii) the ranking of the pathways targeted by a drug using the topology information encoded in the human interactome. We show that PxEA picks up whether drugs target specifically the pathways associated with a disease or common pathways shared across various conditions. We observe that many anti-inflammatory drugs are not specific to the condition they are used for and likely to target pathways involved in the autoimmune endophenotypes.

To further explore shared disease mechanisms for repurposing drugs, we use PxEA and rank drugs for their therapeutic potential in targeting the common disease pathology between T2D and AD. We identify orlistat, a semisynthetic derivative of lipstatin that inhibits lipase—a pancreatic enzyme that breaks down fat—as the top repurposing candidate. Orlistat inhibits hydrolysis of triglycerides, which in turn, reduces the absorption of monoaclglycerides and free fatty acids [34]. Recent evidence indicates that perturbations in unsaturated fatty acid metabolism are tightly coupled to neuritic plaque and neurofibrillary tangle formation in AD patients [35]. Thus, orlistat might help slowing down the plaque and tangle formation due to its effect on the fatty acid metabolism. Targeting of fatty acid metabolism for improving the cognitive performance presents a novel therapeutic approach and is further supported by experiments in mouse models [36].

PxEA can suggest rather counter-intuitive repositioning opportunities such as the use of clenbuterol, an asthmatic drug, in the treatment of metabolic and neurodegenerative diseases such as T2D and AD. In fact, the potential use of clenbuterol in these diseases is not too far fetched: it enhances cognitive performance in aging rats and monkeys [37], improves memory deficit in mice [38], and reduces the insulin resistance in obese rats [39]. On the flip side, while PxEA provides a cellular network based perspective to recommend drugs, it does not take into account dosage-related effects of drugs, potential adverse events, or the genetic background of the patients. For instance, practolol, a beta-adrenergic antagonist that stands out among the T2D-AD candidates, has been withdrawn from the market due to its high toxicity, limiting its potential therapeutic use in the clinical setting. Despite the limitations of PxEA, such as the incompleteness in the drug target, disease and pathway genes, lack of consideration of dosage-related effects or genetic heterogeneity, we believe PxEA is the first step towards achieving endopharmacology, that is, targeting endophenotypes involved across multiple diseases.

## 4. Materials and Methods

### 4.1. Protein Interaction Data and Interactome-Based Proximity

To define a global map of interactions between human proteins, we obtained the physical protein interaction data from a previous study that integrated various publicly available resources [16]. We downloaded the supplementary data accompanying the article to generate the human protein interaction network (interactome) containing data from MINT [40], BioGRID [41], HPRD [42], KEGG [43], BIGG [44], CORUM [45], and PhosphoSitePlus [46]. We used the largest connected component of the interactome in our analyses, which covered 141,150 interactions between 13,329 proteins (represented by ENTREZ gene ids).

Network-based proximity is a graph theoretic approach that incorporates the interactions of a set of genes (i.e., disease genes or drug targets) with other proteins in the human interactome and contextual information as to where the genes involved in pathways reside with respect to the original set of genes [7]. To quantify interactome-based proximity between two gene sets (such as drug targets, pathway genes or disease genes), we used the average shortest path length from the first set to the nearest protein in the second set following the definition in the original study [7]. Accordingly, the proximity from nodes *S* to nodes *T* in a network G(V,E), is defined as

$$d\left(S,T\right)=\frac{1}{∥S∥}\sum_{u\in S}\min_{v\in T}d\left(u,v\right)$$

where d(u,v) is the shortest path length between nodes *u* and *v* in *G*. We then calculated a *z*-score based on the distribution of the average shortest path lengths across random gene sets $S_{random}$ and $T_{random}$ ($d_{random}\left(S,T\right)=d\left(S_{random},T_{random}\right)$) as follows:

$$z\left(S,T\right)=\frac{d\left(S,T\right)-\mu_{d_{random}\left(S,T\right)}}{\sigma_{d_{random}\left(S,T\right)}}$$

where $\mu_{d_{random}\left(S,T\right)}$ and $\sigma_{d_{random}\left(T,S\right)}$ are the mean and the standard deviation of the $d_{random}\left(S,T\right)$, respectively, obtained using 1000 realizations of random sampling of gene sets that match the original sets in size and degree. We refer to the pathways that are significantly proximal (z≤−2) to a disease as the *pathway span* of the disease throughout text.

Note that, instead of average shortest path distances, one can also use random-walk based distances to calculate proximity between gene sets [26]. However, random walks in the networks are inherently biased towards high-degree nodes [47,48] and require additional statistical adjustment [26,48]. Sampling based on size and degree matched gene sets has been shown to be robust against data-incompleteness in the interactome and in the known pathway annotations [7,48].

**Figure 6:**
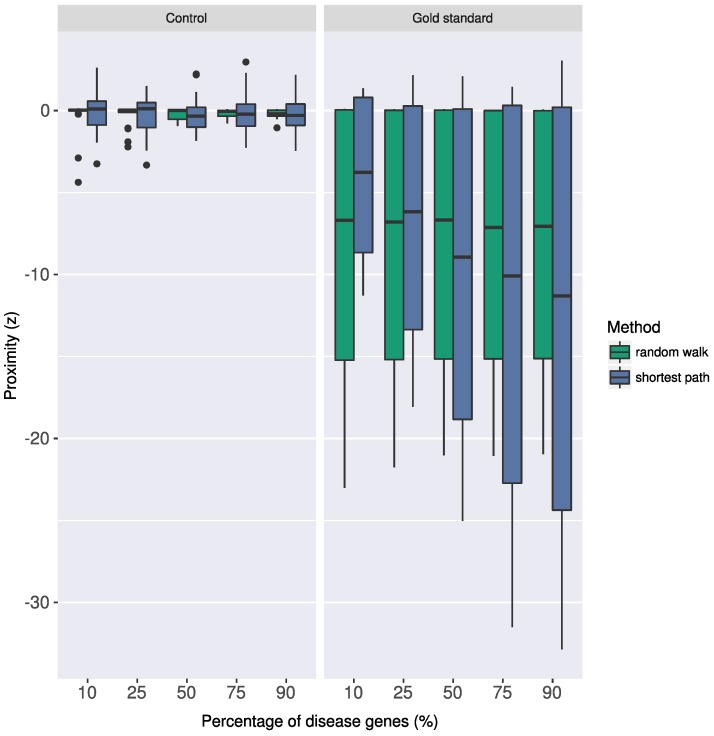
Effect of noise in the pathway data on the random walk and shortest path based proximity calculation. To assess the robustness of the interactome-based proximity in regards to noise in the pathway data, we generated synthetic gold standard pathways containing a certain proportion (*k*%) of the known disease genes in T2D and AD (see text for details). We compared the proximity between these gold standard pathways and the disease genes to the proximity between the control pathways (random groups of gene in the interactome) and the disease genes. The proximity values using random walk and the shortest path for increasing *k* values are shown for the control and gold standard pathways.

To investigate the effect of noise in the pathway data, following the procedure proposed in [49], we created a synthetic pathway data set, in which we defined pathways using a certain percentage *k* of known disease genes in T2D and AD (*k* = 10, 25, 50, 75, 90). Hence, for each value of *k*, we created 10 groups of genes, containing a random sampling of *k*% of the T2D-associated genes. We repeated the procedure using the AD-associated genes, yielding 100 gold standard pathways (10 for each disease across 5 different values of *k*) that were subsets of the known disease genes. For each gold standard pathway, we then generated so called control pathway, that is, randomly selected group of genes in the interactome that match the size of the gold standard pathway under consideration. Next, we assessed the shortest path distance based proximity between the gold standard pathways and the disease genes (proximity of the gold standard T2D pathways to the T2D disease genes and of the gold standard AD pathways to the AD disease genes) and compared it to the proximity of the control pathways to the same disease genes. We also calculated the proximity using random walk scores as proposed in a previous study [50]. We used the random walk implementation in GUILD software package [51] with the default parameters. As one would expect, the gold standard pathways were significantly more proximal (z≤−2) to the disease genes than the control pathways using both proximity calculation approaches (Figure 6). On the other hand, the shortest path distance based proximity distinguished better the overlap between the gold standard pathway genes and the disease genes by providing lower values than the random walk based proximity as the noise in the pathway information decreased (higher values of *k* in the gold pathways).

### 4.2. Disease-Gene, Drug and Pathway Information

We compiled genes associated with nine autoimmune disorders listed in Table 4 using disease-gene annotations from DisGeNET [52]. We downloaded curated disease-gene associations from DisGeNET that contained infromation from UniProt [53], ClinVar [54], Orphanet [55], GWAS Catalog [56] and CTD [57]. To ensure that the disease-gene associations were of high confidence, we kept only the associations that were also provided in a previous large-scale analysis of human diseases [16].

We retrieved drug target information from DrugBank for 1489 drugs in the version 5.0.6 of the database [58], 1466 of which had at least a target in the interactome. UniProt ids from DrugBank were mapped to ENTREZ gene ids using UniProt id mapping file (retrieved on October 2017). We used drug indication information from Hetionet (compound treats or palliates disease edges) that compiled data from publicly available resources [27]. We focused on 78 drugs that were indicated for nine autoimmune disorders above. We created a subset of drugs used for two or more of the autoimmune disorders, yielding 25 drugs across seven conditions (there were no indications for celiac disease, and the two drugs used for Graves’ disease were not used in any other disease).

The ENTREZ gene ids of the proteins involved in biological pathways were taken from the version 5.0 of MSigDB curated gene sets [59]. In our analysis, we used 674 Reactome [60] pathways and the genes associated with these pathways in the MSigDB.

### 4.3. Genetic, Phenotypic and Functional Relationships across Diseases

To identify relationships across disease pairs (autoimmune diseasome), we used the similarities between diseases in terms of the genes and symptoms they share. We assessed the significance of the overlap between genes (or symptoms) associated with two diseases using Fisher’s exact test. An alpha value of 0.05 was set to deem the connections significant (two-sided test p≤0.05). The disease symptom information was taken from a previous study based on text mining of PubMed abstracts [61]. In this study, the number of times a symptom appears in a PubMed abstract was adjusted by the frequency of the symptom in the whole corpus using time frequency-inverse document frequency approach (TF-IDF). To ensure that the disease-symptom associations are of high quality, we considered associations with TF-IDF score higher than 3.5 as suggested in the original study.

Comorbidity relationships across diseases were inferred using data from medical insurance claims, where we assessed whether two diseases occurred more often in the same patient compared to the rest using the relative risk score [62]. Relative risk score relies on the relative occurrence frequencies of diseases across patients, adjusting for the prevalence of the diseases. We mapped the ICD9 codes to MeSH identifiers using the annotations provided by Disease Ontology [63] and we considered the disease pairs with a relative risk score higher than 1 as potential commorbidity links.

To identify pathways enriched in diseases, we used the significance (i) of the overlap between the pathway and disease genes assessed by a one-tailed Fisher’s exact test and (ii) of the proximity between the pathway and disease genes in the interactome. We considered the pathways that had p≤0.05 and z≤−2, respectively, as the pathways that were enriched in a given disease using the two approaches. The pathway information was taken from Reactome and the proximity was calculated as explained above.

### 4.4. PxEA: Proximal Pathway Enrichment Analysis

Toward the goal of pathway level characterization of the common pathology of diseases and to evaluate the therapeutic potential of drugs based on their impact on the common pathways, we developed **P**ro**x**imal pathway **E**nrichment **A**nalysis (PxEA), a novel method that scores drugs based on the proximity of drug targets to pathway genes in the interactome. PxEA uses a GSEA-like running sum score [28], where the pathways are ranked with respect to the proximity of drug targets to the pathways and each pathway is evaluated to see whether or not it appears among the pathways of interest (e.g., common pathways between two diseases). Given *D*, the pathways ranked with respect to their proximity to drug targets, $p_{i}$, the pathway in consideration within *D*, and *C*, the set of pathways of interest, the running score is defined as follows [64]:

$$ES\left(D,C\right)=\sum_{p_{i}\in D}X_{i}$$

where,

$$X_{i}=\{\begin{array}{cc} \sqrt{\frac{\left|D|-|C\right|}{\left|C\right|}}, & if\;p_{i}\in C\\ \;-\sqrt{\frac{\left|C\right|}{\left|D|-|C\right|}}, & otherwise \end{array}$$

To calculate *p*-values for the case study, we repeat the procedure above 10,000 times, shuffling randomly *D* to calculate the expected enrichment score $ES(D^{random},C)$. We then calculate the *p*-value for the enrichment using

$$P=\frac{|ES\left(D,C\right)<ES\left(D^{random},C\right)|}{10,000}$$

The *p*-values were corrected for multiple hypothesis testing using Benjamini-Hochberg procedure [65].

### 4.5. Implementation Details and Code Availability

We used the toolbox Python package for running PxEA, available at github.com/emreg00/toolbox. The proximity was calculated using networkx package that implements Dijkstra’s shortest path algorithm. The statistical tests were conducted in R (www.R-project.org) and Python (www.python.org). The network visualizations were generated using Cytoscape [66] and the plots were drawn using either Seaborn python package [67] or ggplot2 R package [68].

## Figures and Tables

**Table 1:** Number of pathways enriched across nine autoimmune disorders based on the overlap between the pathway and disease genes (one-sided p≤0.05, assessed by a Fisher’s exact test) and the proximity of the pathway genes to the disease genes in the interactome (z≤−2, see Methods for details).

Disease	# of Pathways	
	Overlap	Proximity
celiac disease	7	143
Crohn’s disease	5	116
diabetes mellitus, insulin-dependent	16	121
Graves’ disease	3	92
lupus erythematosus, systemic	17	98
multiple sclerosis	12	138
psoriasis	5	50
rheumatoid arthritis	55	17
ulcerative colitis	6	138

**Table 2:** Pathways shared by autoimmune disorders based on the overlap and proximity of genes (only pathways that appear most commonly across diseases are shown).

Pathway	# of Shared Diseases	
	Overlap	Proximity
interferon gamma signaling	5	8
costimulation by the CD28 family	5	7
cytokine signaling in immune system	5	7
translocation of ZAP-70 to immunological synapse	5	6
phosphorylation of CD3 and TCR zeta chains	5	6
PD1 signaling	5	4
IL-6 signaling	4	8
generation of second messenger molecules	4	6
TCR signaling	4	6
signaling by ILs	3	9
immune system	3	7
downstream TCR signaling	3	7
interferon signaling	3	7
adaptive immune system	3	3
regulation of KIT signaling	2	7
IL-7 signaling	2	6
CTLA4 inhibitory signaling	2	5
chemokine receptors bind chemokines	2	3
extrinsic pathway for apoptosis	2	3
MHC class II antigen presentation	2	2
IL receptor SHC signaling	-	9
IL-3, 5 and GM CSF signaling	-	9
signaling by the B cell receptor BCR	-	8
regulation of IFNG signaling	-	8
growth hormone receptor signaling	-	8
IL-2 signaling	-	8
regulation of signaling by CBL	-	8

**Table 3:** Top ten drug repurposing opportunities to target common T2D and AD pathology, where the drugs that target the same proteins according to DrugBank are grouped together in the same row and the Anatomical Therapeutic Chemical (ATC) classification and indication information within the same group is marked with the first letter of the drug in the parenthesis (if applicable).

Drug	ATC	Hetionet Indication	DrugBank Indication	PxEA Score	Adjusted *p*-Value
orlistat	A08	obesity, type 2 diabetes	obesity	94.07	<0.0001
obeticholic acid, chenodeoxycholic acid	A05	primary biliary cirrhosis (C)	liver disease (O), primary biliary cholangitis (O), gallbladders (C)	74.06	<0.0001
esmolol, practolol	C07	hypertension (E)	atrial fibrillation (E), noncompensatory sinus tachycardia (E), cardiac arrhythmias (P)	70.55	<0.0001
clenbuterol	R03	-	asthma	70.44	<0.0001
erythrityl tetranitrate	C01	-	angina	70.32	<0.0001
fenoterol, arbutamine, bupranolol	R03 (F), G02 (F) C01 (A), C07 (B)	-	asthma (F), coronary artery disease (A), hypertension (B), tachycardia (B), glaucoma (B)	68.97	<0.0001
dalfampridine	N07	multiple sclerosis	multiple sclerosis	68.44	<0.0001
magnesium sulfate	D11, V04, A06, B05, A12	-	eclampsia, acute nephritis, acute hypomagnesemia, uterine tetany	68.27	<0.0001
roflumilast, crisaborole	R03 (R)	chronic obstructive pulmonary disease (R)	chronic obstructive pulmonary disease (R), dermatitis (C), psoriasis (C)	66.33	<0.0001
montelukast	R03	chronic obstructive pulmonary disease, asthma, allergic rhinitis	asthma	65.94	<0.0001

**Table 4:** Disease-gene associations for the nine autoimmune disorders used in this study.

Disease	# of Genes	Genes
celiac disease	11	IL21 CCR4 HLA-DQA1 BACH2 RUNX3 ICOSLG SH2B3 CTLA4 MYO9B ZMIZ1 ETS1
Crohn’s disease	19	DNMT3A IL12B IRGM IL10 CCL2 FUT2 SMAD3 TYK2 ATG16L1 BACH2
		IL2RA NKX2-3 PTPN2 NOD2 TAGAP MST1 DENND1B IL23R ERAP2
diabetes mellitus, insulin-dependent	18	IL10 GLIS3 HLA-DQA1 HLA-DRB1 PTPN22 SLC29A3 INS BACH2 CLEC16A
		PAX4 HLA-DQB1 IL2RA CD69 IL27 HNF1A CTSH SH2B3 C1QTNF6
Graves’ disease	4	RNASET2 CTLA4 FCRL3 TSHR
lupus erythematosus, systemic	29	IKZF1 CFB RASGRP3 PDCD1 RASGRP1 DNASE1 HLA-DRB1 PTPN22 ETS1 TNIP1
		FCGR2B TNFSF4 IRF5 C2 PRDM1 PXK TLR5 TREX1 TNFAIP3 SLC15A4 PHRF1
		HLA-DQA1 STAT4 ITGAX ITGAM BLK C4A BANK1 CR2
multiple sclerosis	15	CD58 CD6 IRF8 HLA-DQB1 CBLB HLA-DRA KIF1B IL2RA
		TNFSF14 VCAM1 IL7R HLA-DRB1 CD24 TNFRSF1A PTPRC
psoriasis	15	IL12B TNIP1 LCE3D IL13 IL23R TYK2 HLA-DQB1 HLA-C FBXL19
		ERAP1 TRAF3IP2 TNFAIP3 TNF REL NOS2
rheumatoid arthritis	23	MIF CD40 ANKRD55 HLA-DRB1 PTPN22 RBPJ IL2RA AFF3 CCL21 REL SLC22A4 CCR6
		IRF5 SPRED2 CTLA4 PADI4 TNFAIP3 NFKBIL1 HLA-DQA2 STAT4 IL6 BLK TRAF1
ulcerative colitis	24	IL12B JAK2 ICOSLG IL1R2 LSP1 CXCR2 IL10 IL7R CXCR1 DAP NKX2-3 CARD9 GNA12
		IRF5 PRDM1 HNF4A CCNY SLC26A3 FCGR2A IL23R IL17REL MST1 TNFSF15 CDH3

## Abbreviations

The following abbreviations are used in this manuscript:

AD	Alzheimer’s disease
ATC	Anatomical Therapeutic Chemical
GSEA	Gene set enrichment analysis
PxEA	Proximal pathway enrichment analysis
T2D	Type 2 diabetes
TF-IDF	Time frequency-inverse document frequency approach
